# First-line toripalimab plus chemotherapy versus chemotherapy for advanced esophageal squamous cell carcinoma: A cost-effectiveness analysis

**DOI:** 10.1371/journal.pone.0325808

**Published:** 2025-06-10

**Authors:** Jing-Wen Han, Yu Zhong, Jin Zhong, Wen-Jing Zeng, Li-Jun Sun

**Affiliations:** 1 Department of Pharmacy, the First Affiliated Hospital, Fujian Medical University, Fuzhou, China; 2 Department of Pharmacy, National Regional Medical Center, Binhai Campus of the First Affiliated Hospital, Fujian Medical University, Fuzhou, China; 3 Department of Pharmaceutical Analysis, School of Pharmacy, Fujian Medical University, Fuzhou, China; 4 Department of Traditional Chinese Medicine, School of Medicine, Xiamen University, Xiamen, China; 5 Department of Pharmacy, Xiangya Hospital, Central South University, Changsha, China; 6 Department of Oncology, Molecular Oncology Research Institute, The First Affiliated Hospital, Fujian Medical University, Fuzhou, China; 7 Department of Oncology, National Regional Medical Center, Binhai Campus of The First Affiliated Hospital, Fujian Medical University, Fuzhou, China; 8 Fujian Key Laboratory of Precision Medicine for Cancer, The First Affiliated Hospital, Fujian Medical University, Fuzhou, China; Hokkaido University: Hokkaido Daigaku, JAPAN

## Abstract

**Objectives:**

This study aims to evaluate the cost-effectiveness of toripalimab combined with chemotherapy versus chemotherapy alone as a first-line treatment for advanced esophageal squamous cell carcinoma (ESCC) from the perspective of U.S. healthcare payers.

**Methods:**

A 10-year partitioned survival model was developed using survival data from the JUPITER-06 clinical trial (NCT03829969). Costs included only direct medical expenses, and health utility values were derived from published literature. One-way and probabilistic sensitivity analysis were performed to assess the robustness of the model.

**Results:**

Toripalimab combined with chemotherapy incurred an incremental cost of $64,483.3 and achieved an incremental effectiveness of 0.53 quality-adjusted life-years (QALY) compared to chemotherapy alone, resulting in an incremental cost-effectiveness ratio (ICER) of $122,771.67 per QALY. This ICER is below the willingness-to-pay threshold in the United States ($150,000). The model results were sensitive to the cost of toripalimab and the utility values of both progression-free and progressed disease states.

**Conclusions:**

The findings indicate that toripalimab combined with chemotherapy as a first-line treatment for advanced ESCC in the United States provides a cost-effective benefit in comparison to chemotherapy alone.

## 1. Introduction

Esophageal cancer (EC) is one of the most common malignancies worldwide, ranking seventh in incidence and sixth in mortality among all malignancies, and there were over 600,000 new cases globally in 2020 [1]. Histologically, EC primarily contains esophageal squamous cell carcinoma (ESCC) and esophageal adenocarcinoma. The distribution of these subtypes varies significantly by region. In Asia, ESCC contributes to approximately 85% of all EC cases [2], whereas in the United States, it accounts for about 30% [3]. Owing to the insidious clinical symptoms of early ESCC, patients with EC were predominantly diagnosed with unresectable, advanced, or metastatic ESCC [4]. The prognosis for ESCC patients remains dismal, with a 5-year relative survival rate of approximately 5.2% [4,5]. Currently, first-line treatment strategies for advanced or metastatic ESCC commonly involve combination chemotherapeutic regimens, such as 5-fluorouracil or paclitaxel paired with platinum-based agents. However, the clinical benefits of these treatment options remain limited. Hence, it is urgent to develop novel drugs and strategies for advanced or metastatic ESCC.

Immune checkpoint inhibitors (ICIs) have been approved for the treatment of various tumors [6–9]. Toripalimab, a humanized immunoglobulin G (IgG)_4K_ monoclonal antibody that specifically targets the human PD-1 receptor, has approved by the National Medical Product Administration of China for the treatment of melanoma, nasopharyngeal carcinoma, EC, *etc*. [10–13] Recent studies indicated that compared with mono-chemotherapy, toripalimab in combination with chemotherapy exhibits promising efficacy in the treatment of advanced ESCC, suggesting that ICIs plus chemotherapy may serve as a potential alternative first-line treatment for patients with advanced ESCC [14–17].

JUPITER-06 was a randomized, double-blind, placebo-controlled phase Ⅲ clinical trial (NCT03829969) [16]. This trial explored the efficacy and safety of toripalimab plus chemotherapy (paclitaxel/cisplatin, abbreviated as TTP) compared to placebo plus the same chemotherapy regiment (abbreviated as TP) as a first-line treatment for patients with advanced ESCC. A total of 514 eligible patients with unresectable, advanced, recurrent, or metastatic ESCC were randomly assigned to TPP arm (n = 257) or TP arm (n = 257). The median overall survival (OS) for the TTP group was 17 months, compared to 11 months for the TP group. The median progression-free survival (PFS) was 5.7 and 5.5 months in TTP and TP arms, respectively. The results manifested that TTP obviously improved both OS and PFS in patients with advanced or metastatic ESCC. Despite the meaningful clinical improvements of the TTP regimen, its high costs cannot be overlooked. Therefore, it is necessary to assess the cost-effectiveness of TTP compared to TP treatment. Toripalimab is currently marketed in the United States; however, there is a lack of clinical data from the U.S. patient population. Therefore, we sought to determine whether the drug has a cost-effectiveness advantage after entering the U.S. market by utilizing data from the Asian Jupiter-06 clinical trial. The present study explored the economic impact of implementing TTP regimen as a first-line treatment for advanced ESCC from the perspective of U.S. healthcare payers, so as to optimal health resource allocation.

## 2. Materials and methods

### 2.1 Targeted population

This study employed the demographic details of participants from the JUPITER-06 Phase III clinical trial. The participants were aged ≥18 years (median age: 62–63 years) and had been diagnosed with unresectable, advanced, recurrent, or metastatic esophageal squamous cell carcinoma (ESCC) [16].

### 2.2 Intervention

According to the design and interventions of the JUPITER-06 trial, patients in the TTP group received toripalimab (240 mg per dose, administered intravenously) combined with chemotherapy (cisplatin 75 mg/m^2^, administered intravenously; paclitaxel 175 mg/m^2^, administered intravenously). Toripalimab plus chemotherapy were scheduled at three-week intervals, spanning a total of seven sessions. Patients in the TP group received chemotherapy alone. Chemotherapy was scheduled at three-week intervals, spanning a total of six sessions. Subsequently, patients were transitioned to a maintenance phase, where they received either toripalimab or a placebo as a single-agent therapy. All patients continued treatment until disease progression, unacceptable toxicity, withdrawal of consent, or a maximum of six cycles [16].

### 2.3 Modeling approach

Cost-effectiveness analysis is currently the most common method used by the National Institute for Health and Care Excellence (NICE) to assess the economic justification interventions in advanced or metastatic cancers [18]. In this study, we constructed a partitioned survival model for cost-effectiveness analysis of TTP versus TP treatment strategies for ESCC patients. The partitioned survival model can determine the number and proportion of individuals in each health state through survival curves. Survival data were digitized from the JUPITER-06 survival curves using the Get Data Graph Digitizer software (version 2.26; http://www.getdata-graph-digitizer.com/download.php). We then refitted and generated new OS and PFS curves according to the method described by Hoyle et al. [19]. Kaplan-Meier survival analysis was conducted using R software (version 3.5.1). The distribution functions included Weibull, log-logistic, log-normal, Gompertz, exponential, and gamma [20]. The Akaike information criterion (AIC) and Bayesian information criterion (BIC) were used to assess goodness of fit and the distribution function with the lowest AIC and BIC values was selected for extrapolation to estimate long-term clinical survival outcomes [21] (S1–S4 Figs and S1 Table in S1 File). The median OS, median PFS, and the tail end of the fitted curves derived from this method were consistent with the observed results from JUPITER-06, thereby validating the model.

### 2.4 Model structure

**Fig 1:**
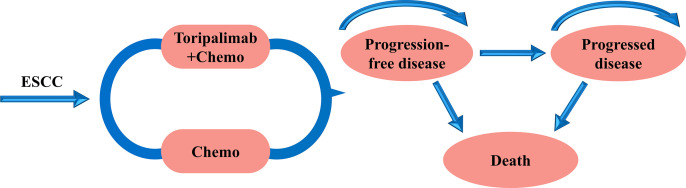
Partitioned survival model simulating the results of the JUPITER-06 trial. All patients started in the PFD state and received appropriate treatment. Patients could enter the PD state and subsequently move to the death state. ESCC: esophageal squamous cell carcinoma.

Patients were assigned to one of three mutually exclusive health states: progression-free disease (PFD), progressed disease (PD), and death. The area above the OS curve was used to estimate the number of patients in the death state. The area under the PFS curve represented the number of patients in the PFD state, while the area between the OS and PFS curves modeled the number of patients in the PD state. The cost-effectiveness analysis was conducted from the perspective of the U.S. healthcare payers. The partitional survival model was developed and analyzed using TreeAge Pro 2020 software (Fig 1). All patients were initially assigned to the PFD state. The model cycle length was set to 3 weeks, corresponding to the treatment cycle. Based on survival data from the JUPITER-06 trial, the time horizon was set to 10 years, sufficient to capture the overall progression of advanced ESCC patients. Before death, patients transitioned between different states, received corresponding treatments, incurred treatment costs, and generated health outcomes. The primary outcome measures included total costs, quality-adjusted life-years (QALY), and incremental cost-effectiveness ratios (ICER), expressed as cost per QALY.

### 2.5 Treatment duration

According to the JUPITER-06 trial, the median treatment duration was 21 weeks for the TTP group and 19.4 weeks for the TP group. Therefore, it was presumed that patients in the TTP and TP groups received corresponding treatments in the PFD state for a maximum of 7 cycles. After disease progression, all patients were assumed to undergo second-line chemotherapy (S2 Table in S1 File). Based on guideline recommendations and systemic treatment information provided by JUPITER-06 trial, second-line chemotherapy regimens for ESCC mainly included nivolumab (240 mg per dose, administered every 2 weeks for up to 5 cycles) [22], pembrolizumab (200 mg per dose, administered every 3 weeks for up to 8 cycles) [23], or single-agent therapies such as docetaxel (75–100 mg/m^2^, every 3 weeks per cycle), paclitaxel (135–250 mg/m^2^, every 3 weeks per cycle), or irinotecan (250 mg/m^2^, every 3 weeks per cycle) [16,24].

### 2.6 Cost input

This study considered only direct medical costs, including drug costs, administration costs, management costs of AEs, and follow-up costs. All drug costs, follow-up costs, administration costs, incidence and management costs of adverse events (AEs), as well as utility model inputs and their sources, are listed in Table 1. It was assumed that patients had equal chances of receiving each second-line treatment after disease progression. Since grade 3 or 4 AEs are expected to result in higher costs and have a greater impact on quality of life, this study only included AEs that were grade ≥3, had an incidence >5%, and showed a difference in incidence rates of ≥3% between groups. The per-cycle cost of AEs management was calculated as: probability of AEs occurrence × cost of AEs management. The costs of AEs were applied only during the first cycle of the model and assumed to occur no more than once per month.

**Table 1:** Model parameters and ranges used in the sensitivity analyses.

Variable	Baseline Value	Range		Reference
**Drug cost per mg, US $**				
Toripalimab	39.318	19.659	47.1816	[26]
Paclitaxel	0.109	0.0872	0.1308	[26]
Cisplatin	2.687	2.1496	3.2244	[26]
Nivolumab	32.296	16.148	38.7552	[26]
Penmbrolizumab	57.603	28.8015	69.1236	[26]
Docetaxel	0.734	0.5872	0.8808	[26]
Irinotecan	0.0987	0.07896	0.11844	[26]
**Drug administration and follow-up, cost per cycle, US $**				
Administration iv, first hour	146.16	116.93	175.39	[27]
Administration iv, additional hour	31.04	24.83	37.25	[27]
Follow-up	70.37	56.3	84.45	[27]
**AEs cost per event, first cycle only, US $**				
Anemia	537	478	585	[28]
Leukopenia	466	415	508	[28]
Neutropenia	466	415	508	[28]
**Risks of serious AEs in TC group (grade ≥ 3)**				
Anemia	0.109	0.0872	0.1308	[16]
Leukopenia	0.202	0.1616	0.2424	[16]
Neutropenia	0.424	0.3392	0.5088	[16]
**Risks of serious AEs in chemotherapy group (grade ≥ 3)**				
Anemia	0.148	0.1184	0.1776	[16]
Leukopenia	0.132	0.1056	0.1584	[16]
Neutropenia	0.331	0.2648	0.3972	[16]
**Utility**				
PFD	0.797	0.6376	0.9546	[25]
PD	0.577	0.4616	0.6924	[25]
**Disutility of serious AEs**				
Anemia	0.073	0.037	0.11	[28]
Leukopenia	0.2	0.15	0.25	[28]
Neutropenia	0.2	0.15	0.25	[28]
**Other parameters**				
Discount rate	0.03	0	0.06	[29]
Body area surface/m^2^	1.8	1.44	2.16	[30]

### 2.7 Utility input

Currently, there is no relevant literature providing health utility values for the PFD and PD states of ESCC. Therefore, this study adopts the health utility values of gastric cancer, which is similar to ESCC, for analysis [25]. Based on the EuroQoL (EQ-5D) responses from the ToGA trial and using the Japanese scoring algorithm, the health utility value for the PFD state was calculated to be 0.797. The health utility value for the PD state was derived from the previous evaluation of GIST by NICE, which was 0.577 [25]. Utility values for adverse events were obtained from the published literature. The utility value for AEs per cycle was calculated as the probability of occurrence of AEs multiplied by their respective utility values. These utility values of AEs were only applied in the first cycle of the model, with the assumption that they occur only once per month.

### 2.8 Cost-effectiveness analysis

A 3% discount rate was applied to both costs and utilities [29]. One-way sensitivity analysis was performed to evaluate the impact of changes in various parameters on the stability of the results. The drug cost baseline value was varied by ±20% as the range of variation. Variation ranges for follow-up costs, AE costs, utilities, discount rate, and body surface area were all derived from published literature. The results were presented using a tornado diagram.

Probabilistic sensitivity analysis was conducted using Second-order Monte Carlo simulations to assess the impact of model parameter uncertainty on the results. All costs were modeled with a Gamma distribution, AE incidence rates, all utilities with a Beta distribution, and body surface area and weight with a normal distribution. Parameters were repeatedly sampled 1,000 times from their respective distributions to evaluate the model. A cost-effectiveness acceptability curve and an incremental cost-effectiveness scatter plot were created to show the probability of each treatment being cost-effective under different willingness-to-pay (WTP) thresholds. The WTP threshold for the United States was set at $150,000, as recommended by Neumann et al [31].

## 3. Results

### 3.1 Base case results

The basic analysis results of this study are shown in Table 2. The total costs for the TTP group and the TP group were $71,153.03 and $6,669.73, respectively, with an incremental cost of $64,483.30. The total utilities generated for the TTP group and the TP group were 1.1 QALY and 0.58 QALY, respectively, with an incremental utility of 0.53 QALY. The ICER was $122,771.67 per QALY.

**Table 2:** The cost and outcome results of the base case analysis.

Treatment	Total cost ($)	Incremental cost ($)	Total effectiveness (QALYs)	Incremental effectiveness (QALYs)	ICER ($/QALY)
TP (Chemo)	6669.73	–	0.58	–	–
TTP (Toripalimab + chemo)	71153.03	64483.3	1.1	0.53	122771.67

### 3.2 One-way sensitivity analysis

**Fig 2:**
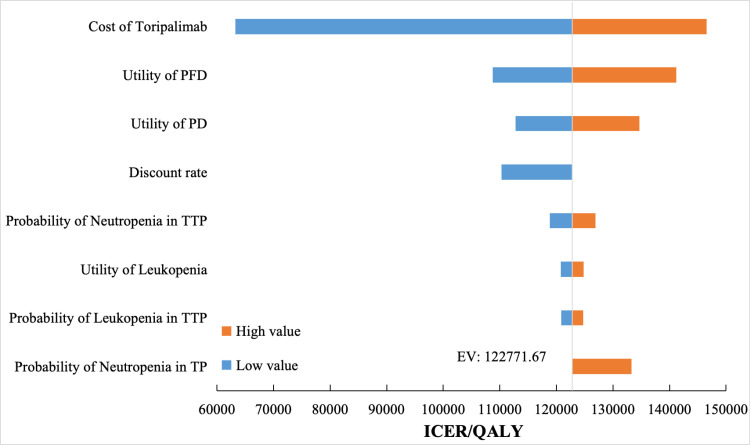
Tornado diagram of one-way sensitivity analysis. The dashed line where the red and blue sections intersect represents the cost per QALY in the base case analysis, which is $122,771.67. TTP: Toripalimab combined with chemotherapy; TP: Chemotherapy; PFD: Progression-free disease; PD: Progressed disease; ICER: incremental cost-effectiveness ratios; QALY: quality-adjusted life-years.

The results of the one-way sensitivity analysis are shown in Fig 2. The cost of toripalimab, as well as the utilities of PFD and PD, were the main factors influencing the ICER. Within the reasonable range of variation (±20%) for these key parameters, the ICER could fluctuate significantly. However, even under the most unfavorable circumstances, all simulated ICER values remained within the set willingness-to-pay threshold, without altering the conclusion that the treatment regimen was cost-effective. Additionally, factors such as the discount rate and the probability of AEs also had a slight impact on the ICER, and the overall model results demonstrated good robustness.

### 3.3 Probabilistic sensitivity analysis

**Fig 3:**
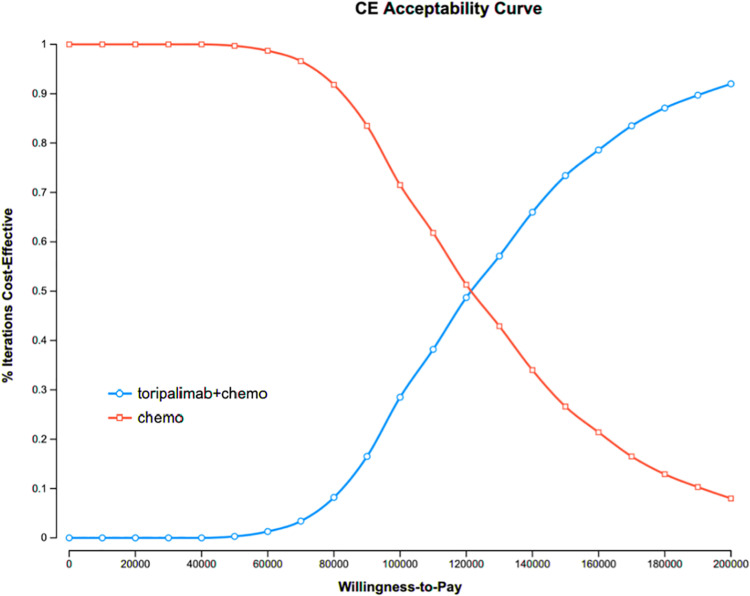
The cost-effectiveness acceptability curves.

**Fig 4:**
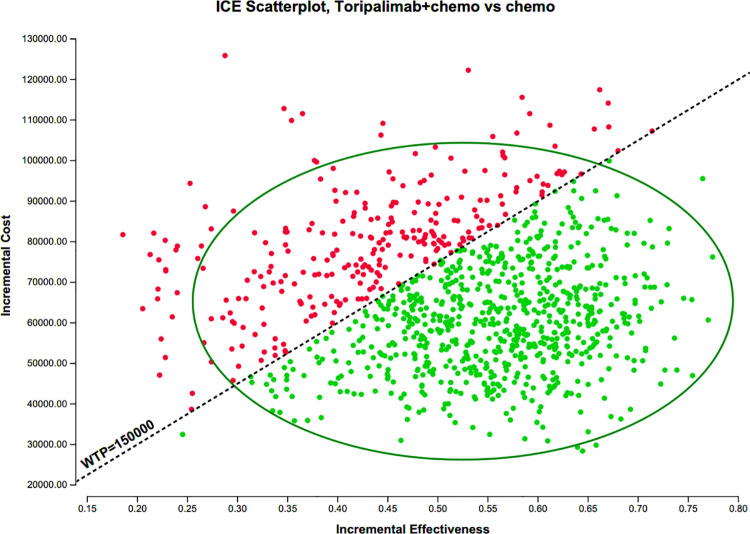
Incremental cost-effectiveness scatter plot of second-order Monte Carlo simulations for 1,000 steps.

The results of the probabilistic sensitivity analysis (Figs 3 and 4) show that at a WTP of $150,000, the probability of the TTP group being cost-effective is 74.8%. If the WTP increases to $200,000, the probability of the TTP regimen being cost-effective rises to 92.5%.

## 4. Discussion

With the advent of immunotherapy, the treatment landscape for advanced ESCC has rapidly evolved into combination therapies based on ICIs, and the clinical outcome of patients with advanced ESCC has significantly altered [32]. According to the JUPITER-06 trial, TTP significantly improves OS and PFS in patients with advanced ESCC. However, new treatment options are often accompanied by high costs. Additionally, no validated predictive molecular biomarkers are currently available to guide the selection of specific treatment regimens [33–35]. The purpose of our study was to evaluate the economic effectiveness of TTP as a first-line treatment for patients with advanced ESCC from the perspective of U.S. healthcare payers to determine the optimal treatment strategy. In the present study, economic analysis results showed that compared with chemotherapy alone, the ICER of patients with advanced ESCC in the TTP group was $122,771.67 per QALY, which was lower than the WTP threshold. The robustness of the results was confirmed by both one-way sensitivity analysis and probabilistic sensitivity analysis. Our findings suggested that TTP may be a cost-effective treatment option for patients with advanced ESCC, and these findings may help guide the selection of personalized treatments for ESCC patients.

Toripalimab received FDA approval in October 2023 for the treatment of advanced nasopharyngeal carcinoma and was officially launched in the U.S. market in January 2024 [36]. However, it remains uncertain whether its use for ESCC can achieve cost-effectiveness by improving patient health benefits, reducing healthcare resource consumption, or both. Currently, there are four published pharmacoeconomic studies evaluating toripalimab combined with chemotherapy as a first-line treatment for advanced esophageal cancer. However, their results varied, and these studies were conducted from the perspective of the Chinese healthcare system [37–40]. This study is the first pharmacoeconomic analysis using an economic modeling approach and the latest evidence to evaluate TTP as a first-line treatment for advanced ESCC patients in the United States. We hope this study can serve as a reference for health insurance policy formulation and clinical decision-making.

Additionally, when the disease progresses, patients may choose various second-line treatments, and survival times in the PD state are inconsistent, making the calculation of treatment costs during PD particularly challenging. Similar economic evaluation studies have often opted to use the average cost of second-line treatments from other studies, overlooking the heterogeneity among patients and failing to accurately reflect the survival outcomes of target patients in the PD state [41–42]. In this study, we carefully considered second-line treatment options based on guidelines, information from randomized controlled trials, and expert clinical opinions. Costs during the PD state were calculated according to the treatment regimens chosen by patients and their survival statuses.

However, our study inevitably has some limitations. First, the patients included in the JUPITER-06 trial were primarily of Asian ethnicity, which may have influenced the results. Due to differences in race, genetic background, environmental factors, and lifestyle, there could be significant disparities in immune response, drug metabolism, disease progression, and resistance mechanisms between Asian and U.S. patients. As a result, efficacy data from Asian populations may not fully reflect the clinical outcomes for U.S. patients. While the clinical data from Asian populations provide an initial basis for evaluation, their applicability in the U.S. market requires further validation through clinical data specific to U.S. patients. Second, there is currently limited research on the pharmacoeconomic and utility values of advanced EC, especially regarding the utility values of PFD and PD states in first-line treatment for advanced ESCC patients. We used the utility values of PFD and PD from first-line treatment for advanced gastric cancer, which is similar to advanced esophageal cancer, as model parameters. Therefore, this method may underestimate or overestimate the actual health status of ESCC patients. Fortunately, the sensitivity analysis demonstrated that the variation of this variable had a minor impact on the model results, which was not sufficient to overturn our study findings. Third, due to difficulties in obtaining the omitted costs (such as direct non-medical costs and indirect costs), we included only direct medical costs in this study. This may affect the results of the overall cost-effectiveness analysis to some extent, especially in cases where the impact of illness on social productivity is significant or where patients rely heavily on supportive care. Fourth, this study only considered grade ≥3 adverse events, and some of the utility values for adverse events were derived from other cancer types.

Although these limitations may restrict the applicability of our study, sensitivity analyses indicated that the utility values of PFD, PD, and AEs had minimal impact on the results. Therefore, this study still serves as a valuable preliminary reference for first-line treatment of advanced ESCC.

## Supporting information

S1 File S1 Fig. The replicated Kaplan-Meier survival curves of overall survival curves for the Group toripalimab plus chemotherapy. S2 Fig. The replicated Kaplan-Meier survival curves of progrssion-free survival curves for the Group toripalimab plus chemotherapy. S3 Fig. The replicated Kaplan-Meier survival curves of overall survival curves for the Group chemotherapy. S4 Fig. The replicated Kaplan-Meier survival curves of Progression-free survival curves for the Group chemotherapy. S1 Table. The fitted survival curve results. S2 Table. Dosage and administration of second-line treatment regimens.
(DOCX)
