# Supplementary material for: First-line toripalimab plus chemotherapy versus chemotherapy for advanced esophageal squamous cell carcinoma: A cost-effectiveness analysis
Source: PLoS One. 2025 Jun 10;20(6):e0325808. doi: 10.1371/journal.pone.0325808 (PMC12151424; doi:10.1371/journal.pone.0325808)
Supplement: S1 File — S1 Fig. The replicated Kaplan-Meier survival curves of overall survival curves for the Group toripalimab plus chemotherapy. S2 Fig. The replicated Kaplan-Meier survival curves of progrssion-free survival curves for the Group toripalimab plus chemotherapy. S3 Fig. The replicated Kaplan-Meier survival curves of overall survival curves for the Group chemotherapy. S4 Fig. The replicated Kaplan-Meier survival curves of Progression-free survival curves for the Group chemotherapy. S1 Table. The fitted survival curve results. S2 Table. Dosage and administration of second-line treatment regimens. (DOCX) [file pone.0325808.s001.docx]

**Supplementary Materials**

**First-line toripalimab plus chemotherapy versus chemotherapy for advanced esophageal squamous cell carcinoma: a cost-effectiveness analysis**

Jing-Wen Han^1,2†^, Yu Zhong^3†^, Jin Zhong^4^, Wen-Jing Zeng^5*^, Li-Jun Sun^6,7,8^^[[1]](#footnote-0)^*

1. Department of Pharmacy, the First Affiliated Hospital, Fujian Medical University, Fuzhou 350005, China

2. Department of Pharmacy, National Regional Medical Center, Binhai Campus of the First Affiliated Hospital, Fujian Medical University, Fuzhou 350212, China

3. Department of Pharmaceutical Analysis, School of Pharmacy, Fujian Medical University, Fuzhou 350122, China

4. Department of Traditional Chinese Medicine, School of Medicine, Xiamen University, Xiamen 361102, China

5. Department of Pharmacy, Xiangya Hospital, Central South University, Changsha, Hunan, China

6. Department of Oncology, Molecular Oncology Research Institute, The First Affiliated Hospital, Fujian Medical University, Fuzhou, Fujian, 350000, China

7. Department of Oncology, National Regional Medical Center, Binhai Campus of The First Affiliated Hospital, Fujian Medical University, Fuzhou, 350212, China

8. Fujian Key Laboratory of Precision Medicine for Cancer, The First Affiliated Hospital, Fujian Medical University, Fuzhou, Fujian, 350000, China


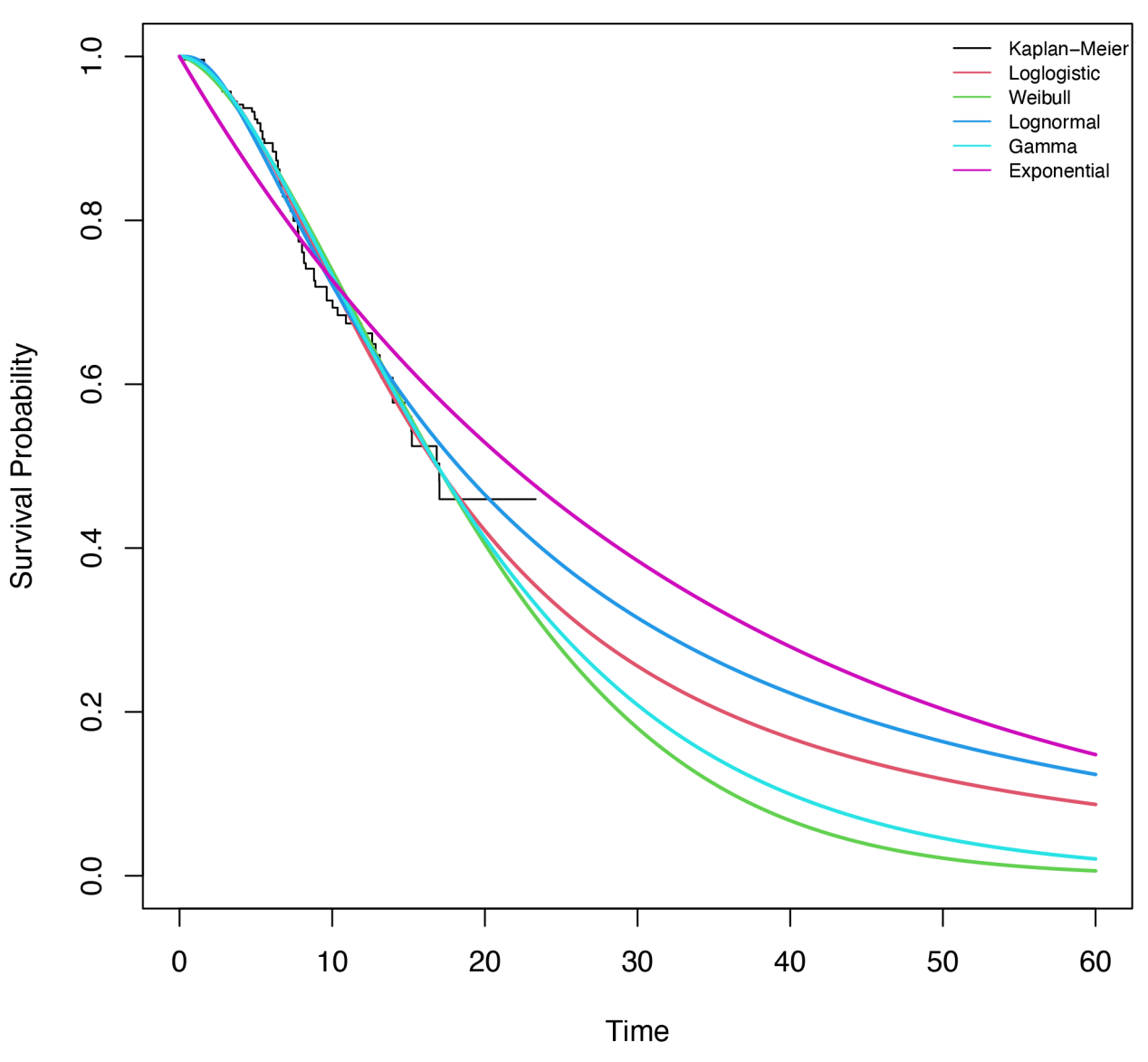


(months)

**S1** **Figure** The replicated Kaplan-Meier survival curves of overall survival curves for the Group toripalimab plus chemotherapy.


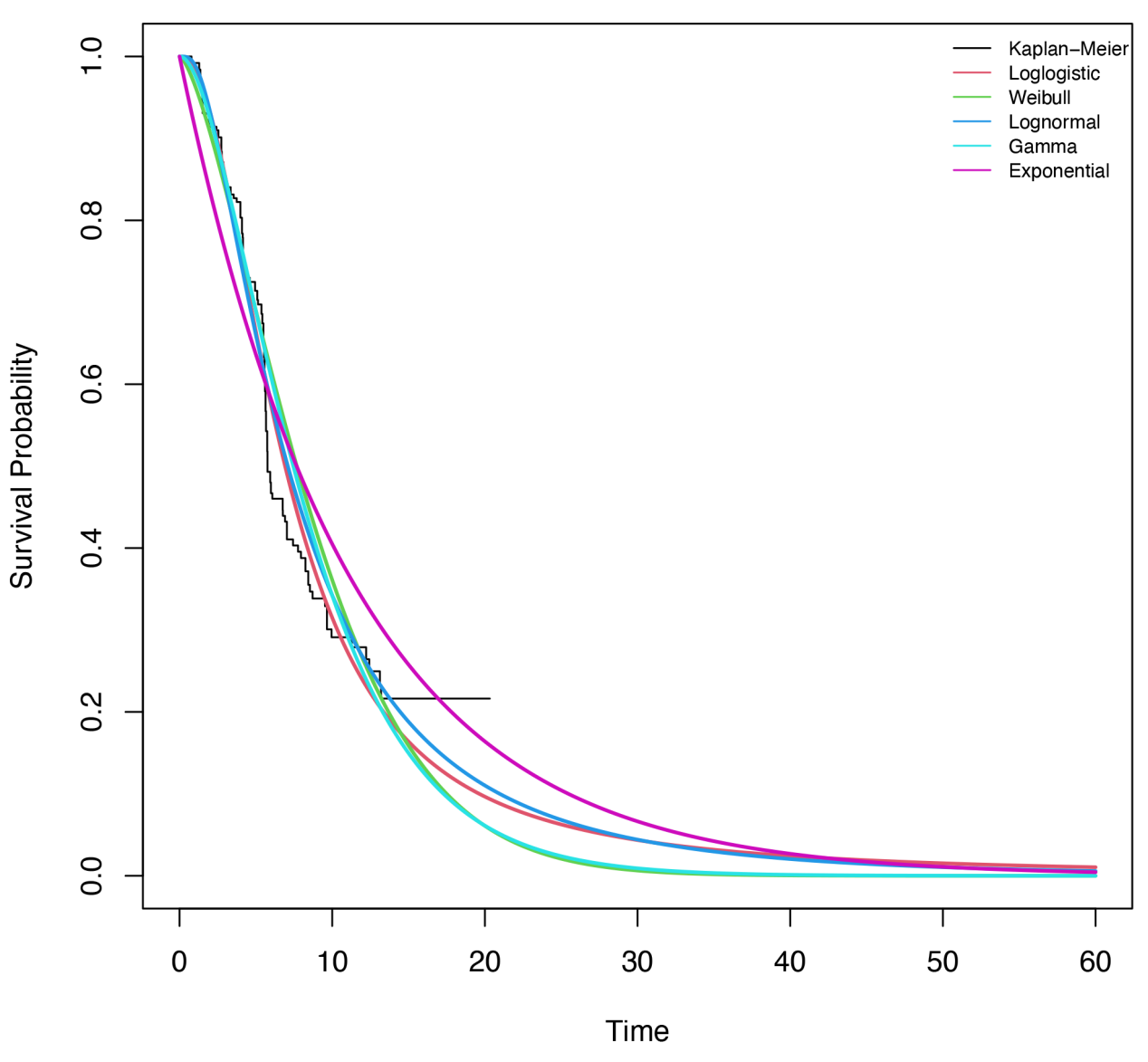


(months)

**S2** **Figure** The replicated Kaplan-Meier survival curves of progrssion-free survival curves for the Group toripalimab plus chemotherapy.


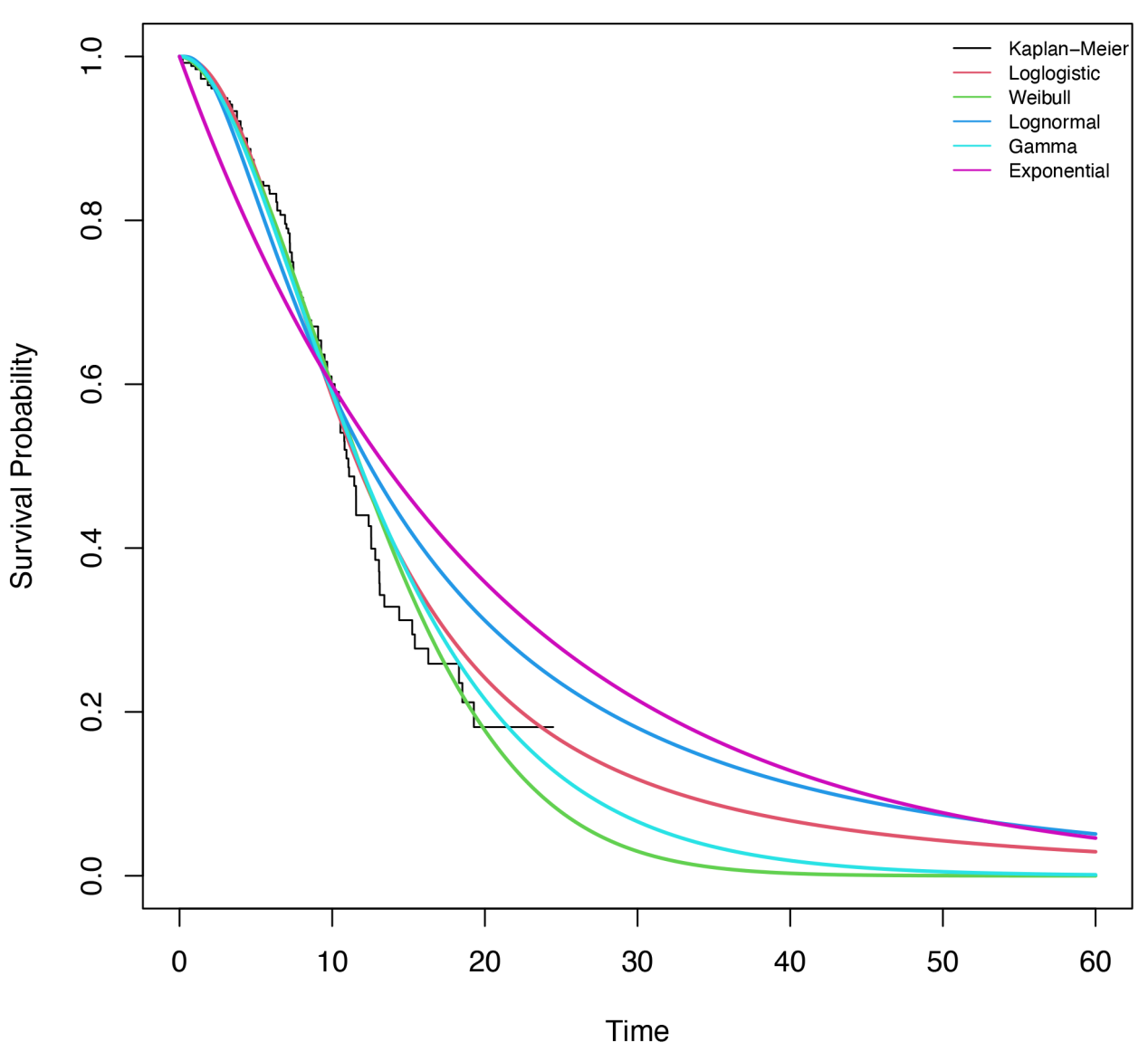


(months)

**S3** **Figure** The replicated Kaplan-Meier survival curves of overall survival curves for the Group chemotherapy.


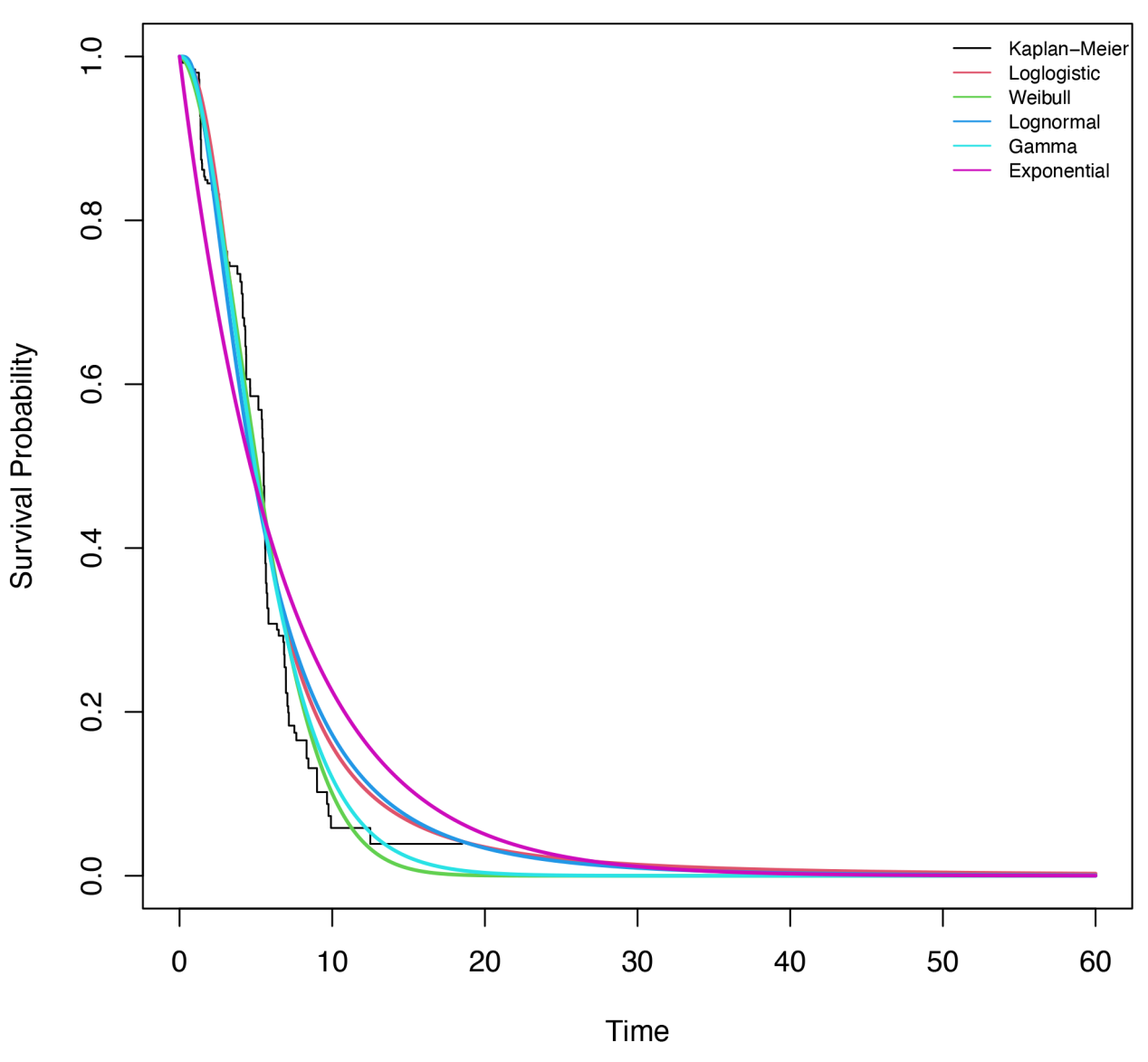


(months)

**S4** **Figure** The replicated Kaplan-Meier survival curves of Progression-free survival curves for the Group chemotherapy.

**S1** **Table** The fitted survival curve results.

| **Model** | **AIC** | | **BIC** | |
| --- | --- | --- | --- | --- |
|  | **Toripalimab + chemo** | **Chemo** | **Toripalimab + chemo** | **Chemo** |
| **OS** | | | | |
| Loglogistic | 622.2420868 | 799.86558 | 629.340239 | 806.963732 |
| Weibull | 625.49737 | 797.157621 | 632.5955222 | 804.255773 |
| Lognormal | 624.518454 | 820.67385 | 631.6166062 | 827.772002 |
| Gamma | 623.7428488 | 799.75487 | 630.841001 | 806.853022 |
| Exponentail | 642.3056308 | 835.679392 | 645.8547069 | 839.228469 |
| Gompertz | 635.2010042 | 806.766902 | 642.2991564 | 813.865054 |
| **PFS** | | | | |
| Loglogistic | 851.556911 | 912.445728 | 858.655063 | 919.54388 |
| Weibull | 875.679967 | 904.340613 | 882.778119 | 911.438765 |
| Lognormal | 852.253633 | 925.556919 | 859.351785 | 932.655071 |
| Gamma | 866.01054 | 905.222003 | 873.108692 | 912.320155 |
| Exponentail | 900.561598 | 971.755119 | 904.110675 | 975.304195 |
| Gompertz | 898.607206 | 933.892927 | 905.705358 | 940.99108 |

**S2** **Table** Dosage and administration of second-line treatment regimens.

| **Drug** | **Dosage and usage instructions** | **Treatment cycle** | **Duration of treatment** |
| --- | --- | --- | --- |
| Camrelizumab | 200 mg, ivgtt, d1 | 2 weeks | 6 cycles [1] |
| Sintilimab | 200 mg, ivgtt, d1 | 3 weeks | 4 cycles [2] |
| Docetaxel | 87.5 mg/m^2^, ivgtt, d1 | 3 weeks | / |
| Paclitaxel | 80 mg/m^2^ , ivgtt, d1,8,15 | 4 weeks | / |
| Irinotecan | 165 mg/m^2^, ivgtt, d1 | 2 weeks | / |

**References**

1. Huang J, Xu J, Chen Y, Zhuang W, Zhang Y, Chen Z, et al. Camrelizumab versus investigator's choice of chemotherapy as second-line therapy for advanced or metastatic oesophageal squamous cell carcinoma (ESCORT): A multicentre, randomised, open-label, phase 3 study. Lancet Oncol. 2020; 21(6):832-842. <https://doi.org/10.1016/S1470-2045(20)30110-8> PMID: 32416073

2. Xu J, Li Y, Fan Q, Shu Y, Yang L, Cui T, et al. Clinical and biomarker analyses of sintilimab versus chemotherapy as second-line therapy for advanced or metastatic esophageal squamous cell carcinoma: A randomized, open-label phase 2 study (ORIENT-2). Nat Commun. 2022; 13(1):857. <https://doi.org/10.1038/s41467-022-28408-3> PMID: 35165274

1. *Corresponding author: E-mail: [29761320@qq.com](mailto:29761320@qq.com) (Li-Jun Sun); [zengwenjing89@csu.edu.cn](mailto:zengwenjing89@csu.edu.cn) (Wen-Jing Zeng).

   Jing-Wen Han^†^ and Yu Zhong^†^ contributed equally to this work. [↑](#footnote-ref-0)
